# ^1^H-NMR and MS Based Metabolomics Study of the Intervention Effect of Curcumin on Hyperlipidemia Mice Induced by High-Fat Diet

**DOI:** 10.1371/journal.pone.0120950

**Published:** 2015-03-18

**Authors:** Ze-Yun Li, Li-Li Ding, Jin-Mei Li, Bao-Li Xu, Li Yang, Kai-Shun Bi, Zheng-Tao Wang

**Affiliations:** 1 Institute of Chinese Materia Medica, Shanghai University of Traditional Chinese Medicine, Shanghai, People’s Republic of China; 2 Department of Pharmacology, Shenyang Pharmaceutical University, Shenyang, People’s Republic of China; Mayo Clinic, UNITED STATES

## Abstract

Curcumin, a principle bioactive component of *Curcuma longa* L, is well known for its anti-hyperlipidemia effect. However, no holistic metabolic information of curcumin on hyperlipidemia models has been revealed, which may provide us an insight into the underlying mechanism. In the present work, NMR and MS based metabolomics was conducted to investigate the intervention effect of curcumin on hyperlipidemia mice induced by high-fat diet (HFD) feeding for 12 weeks. The HFD induced animals were orally administered with curcumin (40, 80 mg/kg) or lovastatin (30 mg/kg, positive control) once a day during the inducing period. Serum biochemistry assay of TC, TG, LDL-c, and HDL-c was conducted and proved that treatment of curcumin or lovastatin can significantly improve the lipid profiles. Subsequently, metabolomics analysis was carried out for urine samples. Orthogonal Partial Least Squares-Discriminant analysis (OPLS-DA) was employed to investigate the anti-hyperlipidemia effect of curcumin and to detect related potential biomarkers. Totally, 35 biomarkers were identified, including 31 by NMR and nine by MS (five by both). It turned out that curcumin treatment can partially recover the metabolism disorders induced by HFD, with the following metabolic pathways involved: TCA cycle, glycolysis and gluconeogenesis, synthesis of ketone bodies and cholesterol, ketogenesis of branched chain amino acid, choline metabolism, and fatty acid metabolism. Besides, NMR and MS based metabolomics proved to be powerful tools in investigating pharmacodynamics effect of natural products and underlying mechanisms.

## Introduction

Hyperlipidemia, a disorder of lipid metabolism, has become one of the public health concerns throughout the world as it could raise the risks of coronary heart disease [1], fatty liver [2], and cancers [3]. Although hypolipidemic chemicals have been developed, their side effects such as diarrhea, nausea, myositis and abnormal liver function are obvious [4]. As an alternative therapeutic approaches, traditional medicines or phytotherapies attract much interest and gain increasing popularity due to their lower toxicity [5].

Curcumin is a principal biological active component of *Curcuma longa* L [6], a medicinal herb used for treatment of biliary disorders, anorexia, coryza, and hepatic disorder in China and other Asian countries [7]. Curcumin were reported to exhibit broad spectral pharmacologic activities including anti-diabetes [8], anti-inflammatory [7], antioxidant [9], cancer-preventive properties [10], and as well. In recent years, anti-hyperlipidemia activity of curcumin attracted increasing attention [9,11], yet the underling mechanism is not well elucidated.

Metabolomics is now a widely utilized bio-analytical methodology in systems biology, providing holistic and unbiased metabolism information associated with diseases or treatments [12,13]. Techniques employed for metabolomics are mainly nuclear magnetic resonance (NMR) and mass spectrometry (MS) [14]. NMR technique is universal (i.e. independent of ionization propensities) and provides most reliable structural and quantitative information of metabolites, but with a lower sensitivity compared to MS [15]. MS technique is selective, sensitive, but the samples cleaning procedures are time consuming. Furthermore, the information obtained by MS is sometimes confusing due to the discriminated ionization ability of different types of compounds and matrix effect [16]. A combination of NMR and MS techniques, offers a powerful methodology for metabolomics studies, showing powerful potentials for pharmacodynamics evaluation and mechanism research [17–19]. Urine metabolomics is most commonly conducted and preferred for metabolomics work due to the advantage that urine can be obtained in large quantities by noninvasive sampling [20,21].

In the present work, serum biochemistry assay and urine metabolomics study combining both NMR and MS techniques were conducted to investigate the intervention effect and metabolism regulations of curcumin on hyperlipidemia mice induced by high-fat diet (HFD), with lovastatin as positive control. Multivariate data analysis, such as principal components analysis (PCA) and orthogonal partial least-squares discriminant analysis (OPLS-DA) were utilized to reveal pharmacodynamics and underlying mechanisms.

## Materials and Methods

### Animals and reagents

C57BL/6Slac mice (4 weeks, male) were obtained from Shanghai SLAC Lab. Animal Co. Ltd. (Shanghai, China) and housed in SPF grade Experimental Animal House at Shanghai University of Traditional Chinese Medicine (Shanghai, China) in environmentally controlled conditions (22°C, a 12-h light/dark cycle with the light cycle from 6:00 to 18:00 and the dark cycle from 18:00 to 6:00) with ad libitum access to standard laboratory chow. Animal treatments were strictly in accordance with the National Institute of Health’s Guidelines regarding the principles of animal care (2004) and approved by the Institutional Animal Care and Use Committee, Shanghai University of Traditional Chinese Medicine (Shanghai, China).

Curcumin (purity > 98%) was provided by Shanghai R&D Centre for Standardization of Traditional Chinese Medicine (Shanghai, China). Lovastatin (purity > 99%) was commercially obtained from Dalian Meilun Biology Technology Co., Ltd. (Liaoning, China).

Acetonitrile and methanol of HPLC grade were purchased from Honeywell Inc. (Morristown, NJ, USA). Formic acid and ammonium acetate of LC-MS grade were obtained from ROE Scientific Inc. (Newark, DE, USA). Deuterium oxide (99.9 atom % D) and leucine enkephalin were obtained from Sigma-Aldrich (St. Louis, MO, USA). Deionized water was purified using a Milli-Q system (Millipore, Bedford, MA, USA). All other chemicals and reagents were of analytical grade and commercially available.

### Treatments

All mice were acclimatized for one week and then randomly divided into five groups (*n* = 6): a control group (Con), a model group (HFD), a positive control group (Lov), two curcumin groups (Cur1 and Cur2). Mice in Con group were fed with a low-fat control diet (D12450B, 10 Kcal% Fat, Research Diets, Inc., New Brunswick, NJ, USA) and the others were fed with a high-fat diet (D12492, 60 Kcal% Fat, Research Diets, Inc., New Brunswick, NJ, USA) for consecutive 12 weeks (accompanying by ca. 25% weight gain). Meanwhile, mice in Lov, Cur1 and Cur2 groups were treated with lovastatin (30 mg/kg), curcumin (40 mg/kg) and curcumin (80 mg/kg), respectively. All drugs were dissolved in pure water. Mice in Con and HFD groups were treated with the same volume of water. Drugs or water was orally administered once a day for 12 weeks.

### Sample collection

After 12 weeks, urine sample of 12 h was collected in metabolism cages. Blood samples were obtained via the arteria cruralis, and then all mice were sacrificed by cervical dislocation. Both blood and urine samples were centrifuged at 4°C at 2, 000 g for 10 min, and the supernatants were stored at -80°C prior to use.

### Serum biochemistry assay

The serum levels of total cholesterol (TC), triglyceride (TG), low-density lipoprotein-cholesterol (LDL-c), and high-density lipoprotein-cholesterol (HDL-c) were assayed using commercially available kits (purchased from Nanjing Jianchen Biotech Inc., Nanjing, China) according to the manufacturer’s instructions. Each sample was assayed in duplicate.

### ^1^H-NMR based Metabolomics analysis

#### Sample preparation

The urine samples were thawed at room temperature, and were centrifuged at 4°C at 2, 000 g for 10 min to remove any sediment. Then 400 μL of the resulting supernatant was mixed with 100 μL of phosphate-buffered saline (0.2 M, pH 7.24), and 50 μL of D_2_O. The final volume was transferred in a 5-mm NMR tube (Norell, Landisville, NJ, USA) prior to analysis.

#### NMR analysis parameters

^1^H-NMR spectra of urines were recorded at 300 K on a Bruker 600-MHz AVANCE III NMR spectrometer (Bruker, Germany), equipped with a 5.0-mm BBO probe, operating at 600.13 MHz for ^1^H. Samples were analyzed using one-dimensional Nuclear Overhauser Effect Spectroscopy (NOESYPR1D, RD-90°-t1–90°-tm-90°-acquire) NMR spectra with water suppression. The mixing time was 100 ms and a total of 64 transients were collected for the spectra. A line-broadening factor of 0.3 Hz was applied to FIDs before Fourier transformation.

#### Data processing

All NMR spectra were phased and baseline-corrected manually using TOPSPIN 3.0 (Bruker, Germany). The spectra were referenced internally to the chemical shift of creatinine at 3.03 ppm. Each ^1^H-NMR spectrum over the ranged 0.0–10.0 ppm was reduced to 250 regions of equal width (0.04 ppm) and the signal intensity in each region was integrated using AMIX (Bruker, Germany). The region of 4.84–5.28 ppm and 5.76–6.00 ppm were removed prior to any statistical analysis in order to eliminate any spurious effect of water suppression or urea. Following removal of these regions, data was normalized in AMIX by dividing each integrated segment by the total area of the spectrum to reduce any significant concentration difference. Output data was imported into SIMCA (version 13.0.3, Umetrics, Umeå, Sweden) for multivariate statistical analysis (pareto-scaled).

#### Statistical analysis

PCA, a classical unsupervised multivariate pattern recognition method, was employed to examine the intrinsic variation within a group and to assess the clustering behavior between groups, showing clear separation among HFD, Con and treated groups (S1 Fig.). Subsequently, OPLS-DA, a supervised pattern recognition method, was further performed to maximize the variation among groups and to determine the variables that contributed to this variation. The qualities of models were validated by determining *R* ^2^ (goodness of fit parameter) and *Q* ^2^ (goodness of prediction parameter) values.

The results were visualized in the form of score plots, where each point represents an individual sample (to show the group clusters), and loading plots or S-plots, where each coordinate represents one ^1^H-NMR spectral region (to identify the variables contributing to the classification). The corresponding variables with variable importance in the projection value (VIP) > 1.0 were chosen as major metabolites, whose intensities were compared to indicate metabolic alterations between groups. Statistical analysis was also performed using one-way analysis of variance (ANOVA) followed by Least-significant difference (LSD) post-hoc test (SPSS, Chicago, IL, USA). A probability of P < 0.05 was considered to be a statistically significant difference between two groups.

#### Identifications of metabolites

Metabolites detected were assigned by comparison with spectra of standard compounds (www.hmdb.ca; www.bml-nmr.org) and the Chenomx NMR software suite (Vers. 7.6, Chenomx, Edomonton, Canada). Additional 2-dimensional NMR experiments were performed for the purpose of confirming chemical shift assignments, including homo-nuclear total correlation spectroscopy (2D ^1^H-^1^H TOCSY) and hetero-nuclear single quantum coherence spectroscopy (2D ^1^H-^13^C HSQC), using standard Bruker pulse programs.

### UPLC-Q/TOF-MS based metabolomics analysis

#### Sample preparation

Urine samples were prepared by mixing 50 μL of urine with 150 μL of 50% (v/v) aqueous methanol. After 20,000 g centrifugation at 4°C for 10 min, a 10 μL aliquot of the supernatants was injected into a Waters UPLC-ESI-Q/TOF-MS system (Waters Corporation, Milford, MA) for analysis.

#### Method development and validation

The urine samples were separated on a Waters ACQUITY^TM^ UPLC-Q/TOF-MS system (Waters Co., Milford, MA, USA) using an ACQUITY UPLC HSS T_3_ column (100 × 2.1 mm i.d., 1.8μm) maintained at 40°C. The mobile phase consisted of A (0.1% formic acid and 5 mM ammonium acetate in water) and B (acetonitrile), using gradient elution: 0–2 min 1–2% B, 2–4 min 2–14% B, 4–16 min 14–24% B, 16–18 min 24–90% B, and finally, the column was re-conditioned with 1% B for 2 min. The flow rate was set at 0.3 mL/min and the injection volume was 10 μL.

The mass spectrometric data were collected on a Synapt G2 quadrupole time-of-flight (Q/TOF) tandem mass spectrometer (Waters, Milford, MA) coupled with an electrospray ionization interface in both positive and negative ion modes (ESI+ and ESI-). The capillary voltage was 3.0 KV (ESI+) or 2.8 KV (ESI-), while the cone voltage was 40 V in both modes. The source temperature was set to 120°C with a cone gas flow of 50 L/h, a desolvation temperature of 350°C and a desolvation gas flow of 650 L/h. The mass spectrometer was set to give 4 eV for low collision energy and a ramp collision energy from 20 to 30 eV. Data were acquired from 80 to 1000 Da and corrected during acquisition using an external reference (lock spray) of leucine enkephalin (*m/z* 556.2771for ESI+; *m/z* 554.2615 for ESI-), infused at a flow rate of 5 μL/min. A scan time of 0.2 s with an inter-scan delay of 0.02 s was used for all analyses.

QC samples were prepared by combining equal aliquots from all urine samples and were injected every six specimens during the whole analysis. QC data obtained was used to assess the stability of the LC/MS platform. For all QCs, six characteristic features were picked out to examine the drift of retention times, *m/z*, and the relative standard deviations (RSD) of the peaks areas. The paired retention time and m/z of these features were 1.69–149.1088 (ESI+), 3.57–330.0604 (ESI+), 17.13–915.7906 (ESI+), 0.81–157.0366 (ESI-), 3.56–328.0453 (ESI-) and 17.63–569.3356 (ESI-). The results proved that variations of retention times were less than 0.2 min, drift values of *m/z* were less than 0.02 Da, and the RSD of peak areas were all below 10% (S1 Table). This confirmed that the system was stable during data collection.

#### MS data preprocessing and analysis

Raw data from Q/TOF-MS were analyzed using Micromass MarkerLynx Application Manager Version 4.1 (Waters Co., Milford, MA, USA) for peak deconvolution and peak alignment with the following parameters: initial retention time 0.5 min, final retention time 18 min, mass tolerance 0.02 Da, ion intensity threshold (3,000 counts) and retention time tolerance 0.2 min. The data were combined into a single matrix by aligning peaks with the same mass—retention time pair together from each data file in the data set. The ion intensities of each peak detected (2451 and 785 MS features for ESI+ and ESI- modes) were normalized to the sum of the peak intensities in each sample. After normalization, the data were processed according to the “80% rule”, briefly only variables with values above zero presenting in at least 80% of each group were kept for the following analysis [22]. The two resulting data sets (938 MS features, ESI+; 45 MS features, ESI-) were separately imported into SIMCA to perform PCA and OPLS-DA in the same manner as described in NMR part. The corresponding VIP value was calculated in the OPLS-DA model. A potential metabolic biomarker was selected when its VIP value was more than 1.00. Potential biomarkers were also performed LSD post-hoc test (SPSS, Chicago, IL, USA). A probability of P < 0.05 was considered to be a statistically significant difference between two groups.

#### Identification of the marker metabolites

Identification of the marker metabolites was achieved through a mass-based search followed by manual verification. First, the m/z value of the molecular ion of interest was searched against the Human Metabolome Database (HMDB). Then, the putative identifications were verified by comparing the MS^2^ fragmentations. Part of the metabolites were further identified by reference standards. Biochemical reactions involved were found through KEGG and HMDB.

## Results and Discussion

### Serum biochemical assay

Serum levels of TC, TG, LDL-c, HDL-c were measured and data was shown in Table 1. Comparing HFD with Con group, serum levels of TC, TG, HDL-c, LDL-c were remarkably increased (by 20% to 30%), indicating occurrence of hyperlipidemia. However, after treatments of curcumin or lovastatin, serum lipid profiles were significantly improved: Especially, LDL-c levels in Cur1, Cur2, Lov groups were remarkable reduced compared to HFD group (P < 0.01); TC and TG levels were also decreased to different degrees in treated group compared with HFD group; On the contrary, HDL-c level in Cur1, Cur2, Lov groups were all increased by ca. 5% comparing to HFD group. Epidemiologic observations and clinical trials have consistently documented a positive relation between LDL-_C_ concentrations and cardiovascular diseases (CVD) risk and a negative relation between HDL-_C_ concentrations and CVD risk [23]. Thus the restored serum lipid profile were considered beneficial. Obviously, curcumin or lovastatin treatment partly recovered high serum lipid profile induced by HFD and their anti-hyperlipidemia effects were comparable [24].

**Table 1:** Serum biochemistry test results (*n* = 6).

	TC	TG	HDL-c	LDL-c
**Con**	3.92±0.50	0.37±0.05	2.58±0.28	0.68±0.09
**HFD**	5.09±0.67^a^	0.44±0.08	3.54±0.32^a^	0.89±0.12^a^
**Cur1**	4.78±0.74	0.40±0.07	3.75±0.27	0.73±0.08^b^
**Cur2**	4.56±0.76	0.40±0.08	3.80±0.34	0.71±0.10^b^
**Lov**	4.50±0.73	0.41±0.09	3.79±0.51	0.66±0.07^b^

Note: Con, control group; HFD, hyperlipidemia group; Cur1, curcumin treated group (40 mg/kg); Cur2, curcumin treated group (80 mg/kg); Lov, lovastatin treated group; TC, total cholesterol; TG, triglyceride; LDL-c, low-density lipoprotein-cholesterol; HDL-c, high-density lipoprotein-cholesterol;

^a^P< 0.01 as compared with Con group;

^b^P< 0.01 as compared with HFD group. Statistical analysis was performed using one-way analysis of variance followed by LSD test.

Lovastatin, a 3-hydroxy-3-methylglutaryl coenzyme A (HMG-CoA) reductase inhibitor, can inhibit the synthesis of cholesterol and increase PPARα expression, which has widespread effects on genes associated with mitochondrial fatty acid oxidation [24]. Lovastatin treatment can reduce serum TC, TG and LDL-_C_ levels, while increase HDL-c level in hyperlipidemia state [25,26].

It was reported that anti-hyperlipidemia effect of curcumin (e.g., on cholesterol and gene expression) is similar to that of statins such as lovastatin, and that curcumin mainly acts by reducing liver cholesterol biosynthesis by inhibiting HMG-CoA reductase [8,24,27]. Curcumin may remove lipids from circulation by up-regulating the low density lipoprotein receptor (LDLr) in the liver and HepG2 cells [28,29]. In addition, free radical scavenging activity of curcumin may inhibit lipid peroxidation, and thus contribute to the lipid profile regulation [30]. As a result, decreased serum levels of TC, TG, LDL-c and increased level of HDL-c were detected in the present study.

### ^1^H-NMR based metabolomics analysis

**Fig 1:**
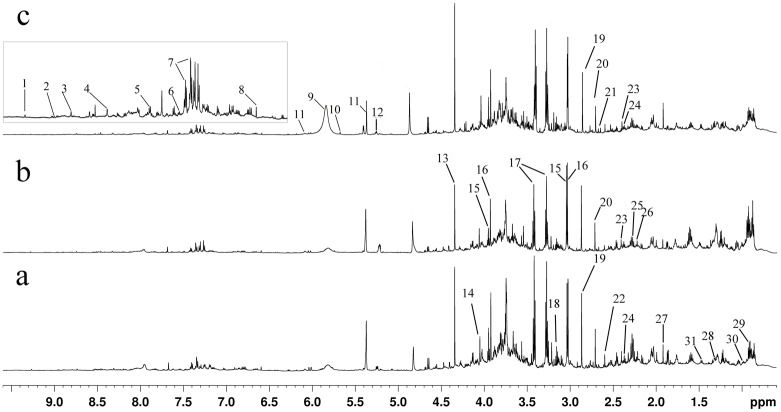
Typical 600 MHz ^1^H-NMR spectra of mice urine from Con (a), HFD (b) and Cur2(c) groups. Metabolites: 1, 1-methylnicotinamide; 2, Niacinamide; 3, Nicotinamide N-oxide; 4, Formate; 5, Hippurate; 6, Benzoate; 7, N-phenylacetylglycine; 8, trans-Aconitate; 9, Urea; 10, cis-Aconitate;11, Allantoin; 12, Glucose; 13, Tartrate; 14, Creatinine; 15, Creatine; 16, Creatine phosphate; 17, Taurine; 18, Choline; 19, Trimethylamine; 20, Dimethylamine;21, Citrate; 22, Methylamine; 23, Succinate; 24, Pyruvate; 25, Acetoacetate; 26, acetone; 27, Acetate; 28, Lactate; 29, Leucine / isoleucine; 30, Valine; 31, Alanine.

**Fig 2:**
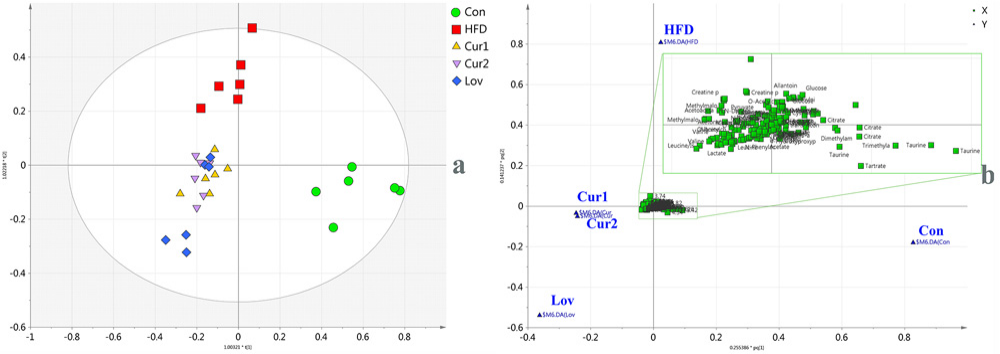
OPLS-DA score plot (a), loading plot (b) of urine ^1^H-NMR spectra obtained from Con, HFD, Lov, GPA1, and GPA2 groups.

Representative ^1^H-NMR spectra of urine from Con, HFD, and Cur2 groups were shown in Fig. 1, with major metabolites identified. The identified metabolites, chemical shifts, and related metabolic pathways were shown in S2 Table. Detail metabolomics differences were revealed by OPLS-DA model (Fig. 2, *R* ^*2*^ *Y* = 0.685, *Q* ^*2*^ = 0.451).

According to score plot of all groups (Fig. 2a), samples of HFD group clustered away from those of Con group, indicating that hyperlipidemia was successfully induced. The treated groups (Cur1, Cur2 and Lov) overlapped each other, deviated from HFD group, and clustered close to the Con group. The distribution suggested that the treatments partially recovered the hyperlipidemia state, and that the anti-hyperlipidemia effect of curcumin (40, 80 mg/Kg) was comparable to that of lovastatin (30mg/kg).

The loading plot (Fig. 2b) revealed the correlations between groups and variables, where variables clustering close to each group were considered to make great contributions to the classification. Accordingly, taurine, citrate, TMA, DMA, tartrate, hippurate, and trans-aconitate were considered to be in high abundance in Con group, while allantoin, creatine, creatinine, glucose, pyruvate, lactate, acetoacetate, acetone, and cysteine were considered to be characteristics of HFD group. Leucine, isoleucine, valine, and alanine were in higher concentrations in treated groups (Cur1, Cur2 and Lov). To detail alteration trends of variables, statistical analysis of major metabolites was performed and shown in Table 2.

**Table 2:** Potential biomarkers detected by NMR and MS, metabolic pathways, as well as fold changes between different groups (*n* = 6).

Metabolites	Detected	HFD/Con	Cur1/HFD	Cur2/HFD	Lov/HFD	Metabolic pathways
**Formate**	NMR	1.11	0.79	0.75	1.16	Folate Metabolism
**Benzoate**	NMR	1.28	0.73	0.65	0.84	Hippurate synthesis
**Hippurate**	NMR	0.73*	1.11	1.16	1.22	Gut microbiome-derived metabolism
**trans-Aconitate**	NMR	0.20	2.79	1.98	2.98	
**cis-Aconitate**	NMR	0.39*	2.35	1.98	3.11*	TCA cycle
**Allantoin**	NMR	1.5	0.58*	0.42**	0.98	Metabolites Related to oxidative stress and kidney damage
**Tartrate**	NMR	0.42**	1.26	1.11	1.48*	
**Cysteine**	NMR	1.03	0.97	0.84	0.79*	Taurine synthesis
**Creatinine**	NMR	1.16	0.97	0.94	0.98	Creatine metabolism
**CP/Creatine**	NMR	1.41**	0.93	0.92	0.9	Creatine metabolism
**Glucose**	NMR	1.15**	0.85*	0.68**	0.67**	Glycolysis and gluconeogenesis
**Taurine**	NMR	0.42**	1.61	2.04	2.19	Bile acid biosynthesis and taurine metabolism
**TMA**	NMR	0.37**	0.73	0.73	1.02	Gut microbiome-derived metabolism
**DMA**	NMR	0.64**	0.87	0.81	0.94	Gut microbiome-derived metabolism
**Citrate**	NMR	0.26**	1.25	1.16	0.95	TCA cycle
**Methylamine**	NMR	1.11	1.22*	1.22*	1.06	Gut microbiome-derived metabolism
**Succinate**	NMR	0.73**	1.08	1.13	1.27*	TCA cycle
**Pyruvate**	NMR	1.12	0.96	0.96	0.93	TCA cycle, glycolysis and gluconeogenesis
**Acetoacetate**	NMR	1.52**	0.89	1.03	0.91	Synthesis and degradation of ketone bodies
**Acetone**	NMR	1.28**	0.95	0.93	0.94	Synthesis and degradation of ketone bodies
**Acetate**	NMR	0.78**	1.28**	1.21**	1.08	Fatty acid oxidation
**Alanine**	NMR	1.23**	1.17*	1.20**	1.22**	Glycolysis and gluconeogenesis
**Lactate**	NMR	1.13	1.20	1.15	1.29	Glycolysis and gluconeogenesis
**MMA**	NMR	1.51**	0.94	0.93	0.91	fat and protein metabolism
**Valine**	NMR	1.34**	1.37**	1.40**	1.38**	Valine, leucine and isoleucine biosynthesis
**Leu/ILE**	NMR	1.29*	1.42**	1.46**	1.43**	Valine, leucine and isoleucine biosynthesis
**Palmitoylglycine**	ESI+	5.96**	0.24**	0.20**	0.20**	Fatty acid metabolism
**Creatine**	ESI+	1.02	0.74*	0.49**	0.86	Creatine metabolsim
**Creatinine**	ESI+	1.20	0.63*	0.31**	0.91	Creatine metabolsim
**Glucose**	ESI+	1.29**	0.79*	0.73**	0.74**	Glycolysis and gluconeogenesis
**Succinate**	ESI+	0.87*	1.05	1.10	1.17*	TCA cycle
**Acetylcarnitine**	ESI+	2.10**	0.55**	1.40**	0.67	Fatty acid metabolism
**3-Hexenedioix acid**	ESI+	2.72**	0.45**	0.40**	0.58**	Fatty acid metabolism
**Cysteine**	ESI+	1.12	0.75*	0.68*	0.82	Taurine synthesis
**Taurine**	ESI-	0.84	1.04*	1.06**	1.12	Bile Acid Biosynthesis
**Cysteine**	ESI-	1.07*	0.85	0.70	0.98	Taurine synthesis

Note: HFD, Con, Cur1, Cur2, Lov represent hyperlipidemia, control, curcumin (40 mg/kg), curcumin (80 mg/kg), lovastatin (30 mg/kg) groups, separately. TMA short for trimethylamine; DMA short for dimethylamine; MA short for methylamine; MMA short for methylmalonate; CP represent creatine phosphate; Leu/ILE short for leucine and isoleucine. XXX/YYY means integral of metabolite in XXX group was divided by that of YYY group. The ratio over 1.00 indicated an increase, while ratio less than 1.00 indicated a decrease. Statistical analysis was performed by one-way analysis of variance followed by LSD test.

*p<0.05;

**p<0.01.

Comparing HFD groups with Con group, the differences in metabolites contents were expected and had been confirmed in previous reports [31]. The metabolism alterations were as follows: in HFD induced animals, synthesis of cholesterol and ketone bodies, gluconeogenesis and glycolysis were enhanced, while tri-carboxylic acid (TCA) cycle was suppressed [31].

**Fig 3:**
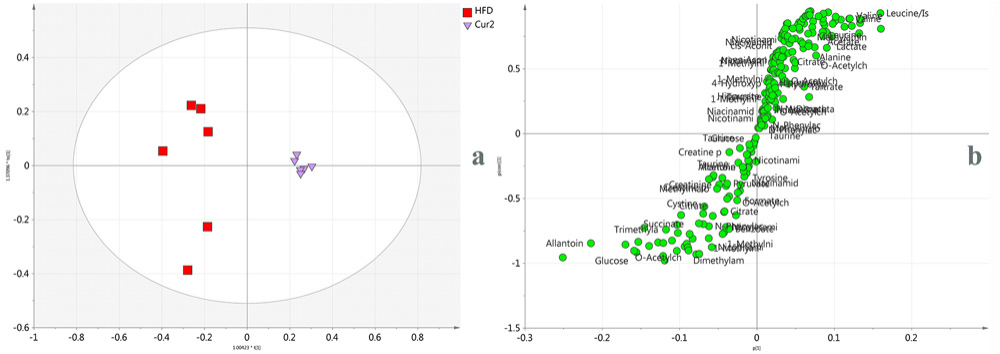
OPLS-DA score plot (a) and S-plot (b) of urine ^1^H-NMR spectra obtained from HFD and Cur2 groups.

To further reveal anti-hyperlipidemia effect of curcumin, OPLS-DA model was carried out between Cur2 and HFD groups. The model fit well with *R* ^*2*^ *Y* value 0.981 and *Q* ^*2*^ value 0.864. The score plot (Fig. 3a) showed good separation, confirming the anti-hyperlipidemia effect. Corresponding S-plot was shown in Fig. 3b, where coordinates in the lower-left quadrant were metabolites significantly increased in Cur2 group compared with HFD group, while those in the upper-right quadrant represent the decreased ones. From the S-plot, metabolites altered in the two groups were selected and discussed.

Branched chain amino acid (BCAA: leucine, isoleucine and valine) levels were increased by ca. 40% in Cur2 (or Lov) group in contrast to HFD group. The increases were consistent with previous report associated with anti-hyperlipidemia effect [32]. It may be inferred that curcumin or lovastatin treatment suppressed the ketogenesis, thus leading to elevated levels of BCAA. Besides, BCAA levels were increased in HFD group compared to control, which were verified previously and BCAA were regarded as hallmarks for hyperlipidemia and insulin resistance [33]. However, relationship between insulin resistance and curcumin treatment remains to be revealed.

Elevated taurine level (by ca. 60%) was noticed after treatment of curcumin. Interestingly, taurine level was formerly decreased by 60% in HFD group compared to Con group. The decrease may be attributed to the increasing conjunction of taurine with bile acid, whose synthesis was up-regulated in hyperlipidemia [34]. Thus elevated taurine levels in Cur2 group indicated that synthesis of cholesterol was suppressed after supplement of curcumin, as had been previously reported [11]. The superposition of Cur1, Cur2 groups with Lov group in the score plot (Fig. 2a) confirmed that curcumin may exert its anti-hyperlipidemia in a similar way to lovastatin [11,27].

Compared with HFD group, levels of ketone bodies (acetone and acetoacetate) were decreased by ca. 10% in Cur2 group, indicating that synthesis of ketone bodies was suppressed by treatment of curcumin. Formerly, synthesis of ketone bodies was significantly increased in HFD group compared with Con group, with acetone and acetoacetate levels elevated by 28% and 52%. The decrease of ketone body synthesis by curcumin was expected and consistent with previous reports associated with anti-hyperlipidemia effect [35], though it had not been recovered to normal level.

The TCA cycle intermediates, including citrate, succinate, and cis-aconitate were all increased in Cur2 group compared with HFD group, suggesting that TCA cycle were up-regulated after treatment of curcumin. Originally, in hyperlipidemia mice TCA cycle was suppressed with intermediates level decreased, which indicated the energy consumption pattern was shifted to lipid oxidation in response to hyperlipidemia [31]. The up-regulation of TCA cycle after treatment of curcumin may suggest the energy consumption recovered.

The lactate, pyruvate and glucose levels in Cur2 group were decreased compared with HFD group (ca. 10%), while alanine level was found increased by ca. 20%. The alterations were associated with glucose metabolism. Consistent with previous reported, in our case of hyperlipidemia excess acetyl-CoA accumulated led to elevated levels of pyruvate, lactate and alanine, due to elevated glycolysis and gluconeogenesis [31,32]. Subsequently, treatment of curcumin or lovastatin decreased urinary levels of pyruvate, lactate, and glucose, implying that glycolysis and gluconeogenesis were suppressed. The elevated alanine level may stem from transformation of acetyl-CoA while gluconeogenesis from amino acids were suppressed.

Allantoin level was noticed to decrease after treatment of curcumin (by ca. 50%). Recognized as a convenient biomarker for oxidative stress [36], the decrease of allantoin was believed to benefit from the antioxidant activity of curcumin. Besides, it was worth to mention that allantoin level was previously increased by a half in HFD group compared with Con group due to increased oxidative stress.

Urinary hippurate level was increased by 10% in Cur2 group compared with HFD group. Whereas the benzoate level was decreased (by 35%). Hippurate, is generated from the conjugation of glycine and benzoic acid [37]. Alterations of hippurate and benzoate levels may reflect variation of the microbial activity and micro floral composition of the colon [38,39]. In addition, hippurate level was reported to decrease in HFD induced obesity mouse due to insulin resistance [40]. Therefore, increased hippurate levels after treatment of curcumin may indicate improvement of insulin resistance.

Decreased urinary levels (ca. 10%) of creatine, creatinine, and creatine phosphate were detected in Cur2 group compared with HFD group. Meanwhile, their levels were noticed to increase in HFD group compared with Con group. Levels of creatine metabolites may reflect injuries of liver and kidney, and elevated levels of them in HFD group may be due to long-term exposure to HFD [31]. While decreases of these metabolites in Cur2 group may indicate that liver and kidney injuries were alleviated [38]. In addition, urinary creatinine levels have been reported to increase in correlation with oxidative stress and to decrease following antioxidant administration [41], it was speculated that the antioxidant activity of curcumin could be a possible explanation for the decrease.

Acetate level was increased (by ca. 20%) in Cur2 group. Acetate is an end product of fatty acid oxidation, and increased urinary excretion may serve to eliminate excess amount of acetyl moieties from fatty acids *beta*-oxidation [42]. Previously, compared with Con group, acetate level was notice to decrease in HFD group, which was consistent with previous report [31].

Choline metabolism was also involved. It was noticed that trimethylamine (TMA) and dimethylamine (DMA) levels were decreased by ca. 20% in Cur2 group compared with HFD group, while methylamine (MA) level was increased by 20%. Previously, it had been reported urinary TMA and DMA levels decreased in hyperlipidemia caused by HFD feeding [43]. Curcumin treatment reversed the increases caused by HFD, indicating partial recovery of choline metabolism. Besides, urinary excretion of methylamines was directly related to gut microbiota metabolism [44]. The alterations indicated gut microbiota metabolism was effected.

### Metabolomics analysis using Q/TOF-MS

**Fig 4:**
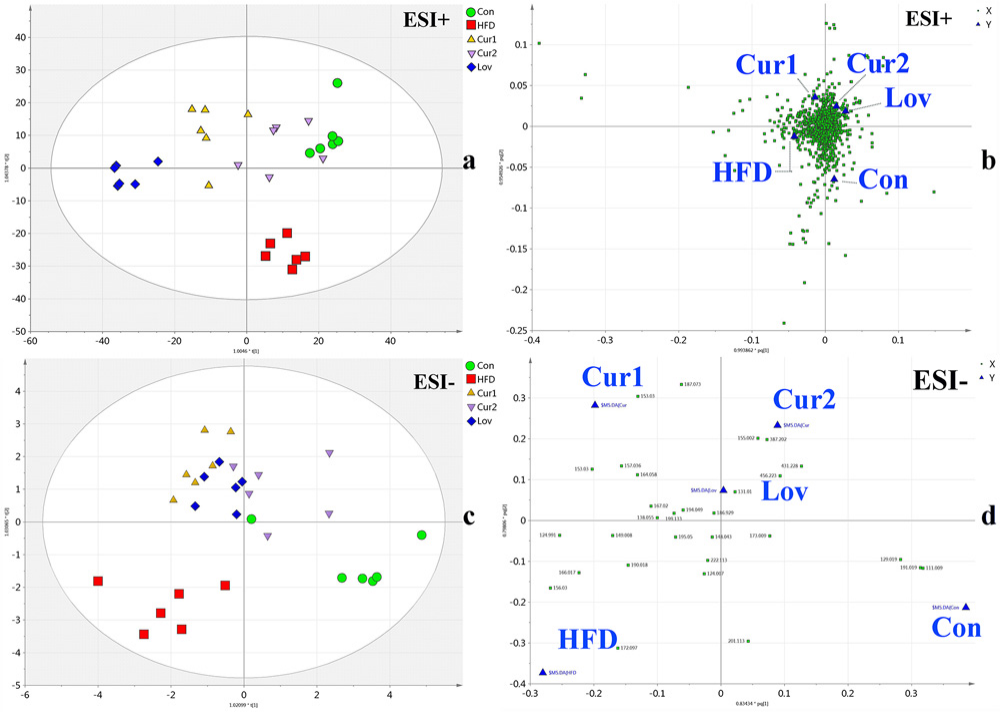
OPLS-DA analysis of UPLC-Q-TOF/MS data of five groups. Score plots in positive and negative modes were labeled as a and c, corresponding loading plots were assigned as b and d.

MS-based metabolomics present similar result with that by NMR, confirming the hypolipidemic effect of curcumin. The representative base peak intensity (BPI) chromatograms of Con, HFD and Cur2 groups were shown in S2 Fig. (ESI+ and ESI-). The MS data acquired was also analyzed by OPLS-DA models (ESI+, *R* ^*2*^ *Y* = 0.833, *Q* ^*2*^ = 0.642; ESI-, *R* ^*2*^ *Y* = 0.691, *Q* ^*2*^ = 0.304). According to the score plots (Fig. 4, a & c), hyperlipidemia was successfully established, with dots representing HFD group distributed away from those of Con group. The treatment of curcumin or lovastatin deviated the dots of treated groups from HFD group to Con group, suggesting the anti-hyperlipidemia effect. Besides, the score plots may reveal that curcumin treatment (80 mg/kg) better recovered the abnormal metabolism than treatment of curcumin (40 mg/kg) or lovastatin (30 mg/kg), with Cur2 group clustering closest to Con group.

Corresponding loading plots (Fig. 4, b & d) were inspected. Metabolites with VIP>1.0 were selected as potential biomarkers responsible for the discriminations (183 for ESI+ and 15 for ESI-). Among them, nine were identified, with retention time, molecular mass, related pathway and postulated identity provided in S3 Table. Some of the metabolites detected by MS have been identified by NMR analysis, and the alterations trends revealed by both techniques were consistent (shown in Table 2).

To further reveal the metabolism regulations, OPLS-DA model was carried out between Cur2 and HFD groups, with score plots and S-plots present in S3 Fig. Comparing Cur2 group with HFD group: leucine, isoleucine and succinate levels were increased, indicating enhanced synthesis of ketogenic amino acid and TCA cycle; while glucose, creatine and creatinine levels were decreased, suggesting creatine metabolism and gluconeogenesis were suppressed; taurine level was increased, indicating reduced taurine consumption and declined synthesis of cholesterol; cysteine level was decreased, which may be attributed to an amelioration of hyperlipidemia state, considering increased plasma total cysteine as a risk factor for atherosclerosis in hyperlipidemia patients [45].

In addition, MS based metabolomics provided more information. It was revealed that urinary palmitoylglycine, acetylcarnitine, and 3-hexenedioic acid levels were increased 2–5 times in HFD group compared with Con group. Nevertheless, after treatment of curcumin or lovastatin, their urinary levels were reduced. Palmitoylglycine is one of acylglycines, which are normally minor metabolites of fatty acids. Elevated levels of acylglycines in urine are associated with various fatty acid oxidation disorders [46,47]. The elevated level urinary palmitoylglycine in HFD group may be induced by fatty acid oxidation disorders. While after the treatment of curcumin, decreased palmitoylglycine level suggested the disorders of fatty acid oxidation were improved. Acetylcarnitine is an acetic acid ester of carnitine that facilitates movement of acetyl CoA into the matrices of mammalian mitochondria during the oxidation of fatty acids. Elevated urinary level of acetylcarnitine was reported with fasting state or diabetic ketoacidosis [48,49]. It was referred elevated excretion of carnitine esters may serve as a mechanism to remove excess organic acids or acyl moieties [50–52]. In HFD group, elevated urinary acetylcarnitine excretion served the same purpose, removing excess acyl moieties and allowing regeneration of free CoA to be used to maintain normal metabolic functions of the mitochondrion [50]. After treatment of curcumin or lovastatin, urinary excretion of acetylcarnitine declined significantly, which indicated the excess amount of acyl moieties were eliminated in other ways (up-regulated TCA cycle or elevated excretion of urinary acetate) and functions of the mitochondrion were further enhanced [11,27]. 3-Hexenedioic acid is an unsaturated dicarboxylic acid metabolite, which has been reported with increased excretion in patients with dicarboxylic aciduria caused by fatty acid metabolism disorders [53,54]. The urinary excretion of 3-hexenedioic acid is increased in conditions of augmented mobilization of fatty acids or inhibited fatty acid oxidation [55]. It has been reported that curcumin treatment up-regulated gene related with fatty acid oxidation [11]. Thus curcumin or lovastatin treatment decreased urine level of 3-hexenedioic acid, leading effective mobilization of fatty acids.

### Metabolic pathways and Drawbacks of the metabolomics work

**Fig 5:**
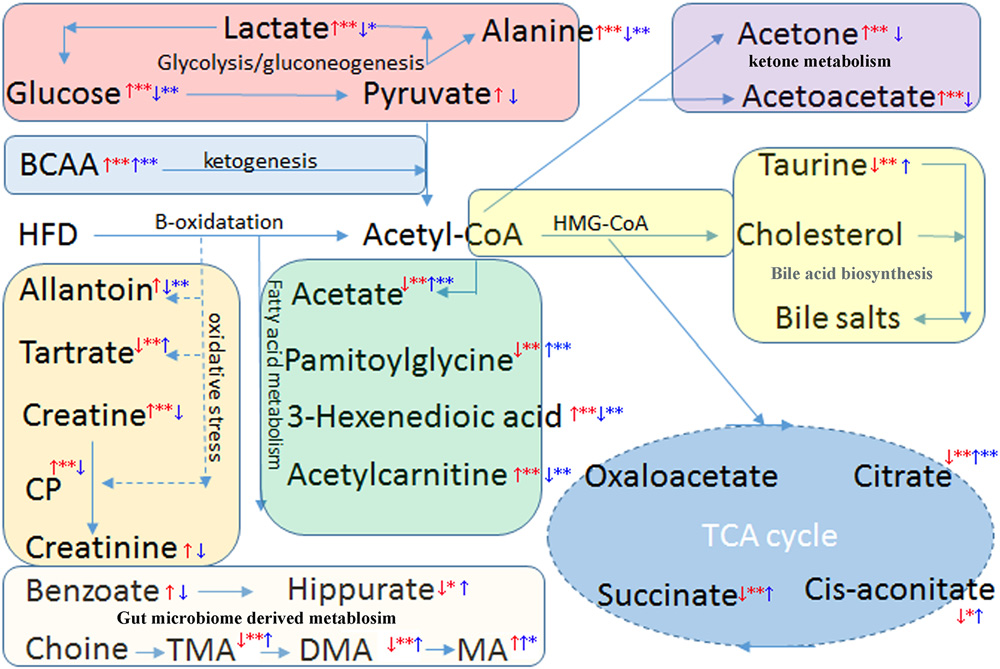
Potential metabolic pathways disturbed in hyperglycemia mice induced by HFD and alterations by curcumin treatment.

In summary, curcumin treatment partially recovered the metabolism disorders induced by HFD and exerted good anti-hyperlipidemia effect. The metabolic pathways included were proposed as follows: TCA cycle, glycolysis and gluconeogenesis, synthesis of ketone bodies and cholesterol, ketogenesis of BCAA, choline metabolism, and fatty acid metabolism (Fig. 5).

However, it has to be confessed that the present work was defective due to the absence of serum metabolomics analysis, which may provide additional convincing data. Besides, a time course based dynamic metabolomics study will be conducted in the future to identify more detailed metabolism profiles.

## Conclusions

In the present work, anti-hyperlipidemia effect of curcumin was confirmed by both serum biochemistry and metabolomics analysis. The anti-hyperlipidemia effect of curcumin was comparable to that of lovastatin. We employed NMR and MS based metabolomics approaches to reveal metabolism regulations of curcumin on hyperlipidemia mice induced by HFD feeding. It turned out that curcumin treatment can partially recover the metabolism dysfunction caused by hyperlipidemia via the possible metabolic pathways: TCA cycles, *beta*-oxidation of fatty acids, synthesis of ketone bodies and cholesterol, ketogenesis of BCAA, glycolysis and gluconeogenesis, creatine metabolism, choline metabolism. Through this work, NMR and MS based metabolomics of urine proved the potential capacities in evaluating pharmacological effect of traditional medicines or natural products and revealing holistic regulations on mechanisms.

## Supporting Information

S1 Fig PCA score plot (a), loading plot (b) of urine ^1^H-NMR spectra obtained from Con, HFD, Lov, GPA1, and GPA2 groups. (TIF)

S2 Fig Representative base peak intensity chromatograms of urine samples from Con, HFD, Lov, Cur2 groups in positive and negative ionization modes. (TIF)

S3 Fig OPLS-DA score plot (a, c) and S-plot (b, d) of UPLC-Q-TOF/MS data obtained from HFD and Cur2 groups. a, b for ESI+ mode and c, d for ESI- mode. (ESI+, *R* ^*2*^ *Y* = 0.987, *Q* ^*2*^ = 0.903; ESI-, *R* ^*2*^ *Y* = 0.942, *Q* ^*2*^ = 0.798).
(TIF)

S1 Table Stability of UPLC-Q/TOF MS platform. (DOCX)

S2 Table ^1^H-NMR assignment results of the identified metabolites, chemical shifts and related metabolic pathways. (DOCX)

S3 Table Potential biomarkers identified by MS, as well as chromatographic retention time, measured molecular mass, and related pathway. (DOCX)
