# Supplementary figures and images for: 1H-NMR and MS Based Metabolomics Study of the Intervention Effect of Curcumin on Hyperlipidemia Mice Induced by High-Fat Diet
Source: PLoS One. 2015 Mar 18;10(3):e0120950. doi: 10.1371/journal.pone.0120950 (PMC4364983; doi:10.1371/journal.pone.0120950)

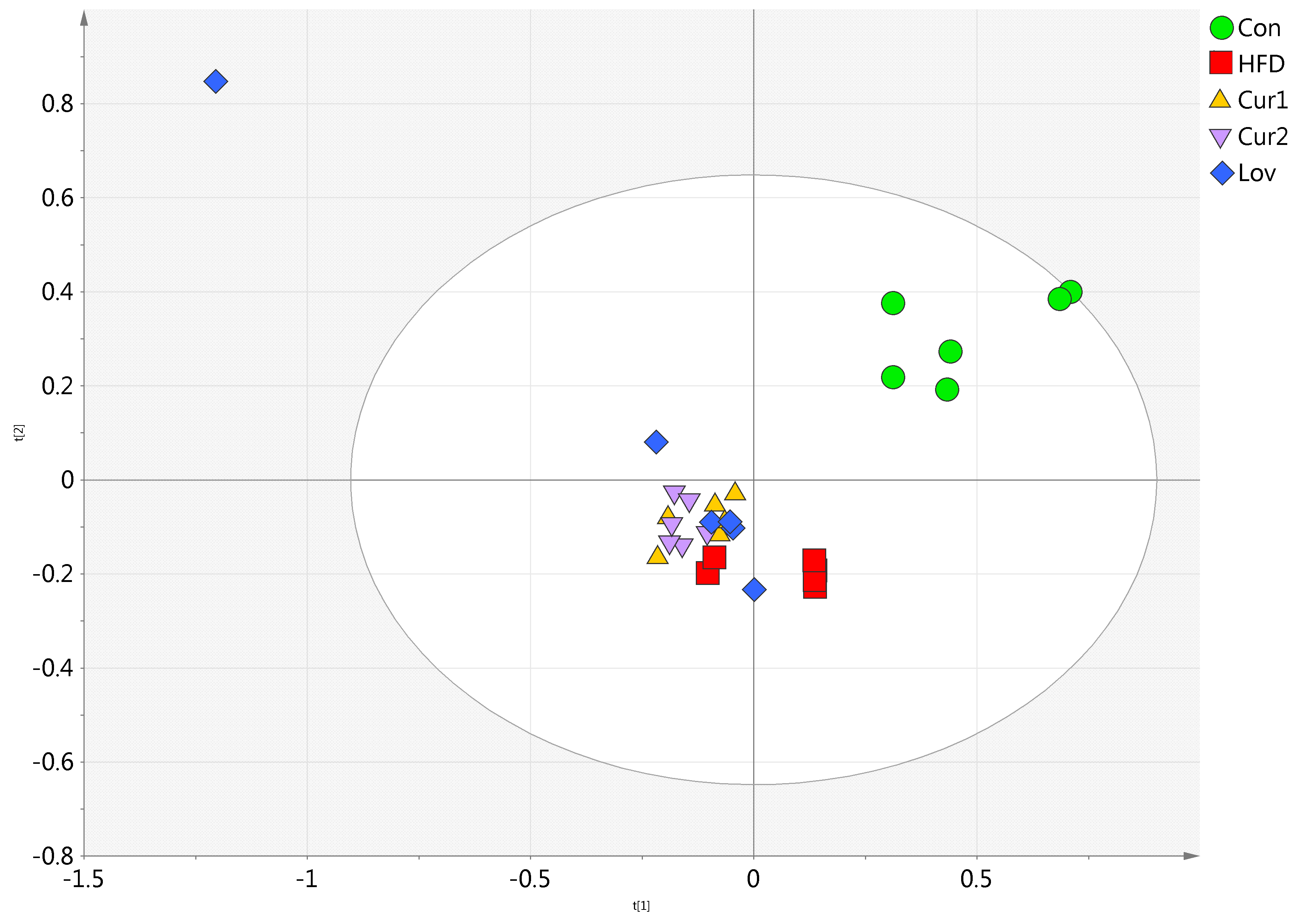

Supplement: S1 Fig — (TIF) [file pone.0120950.s001.tif]

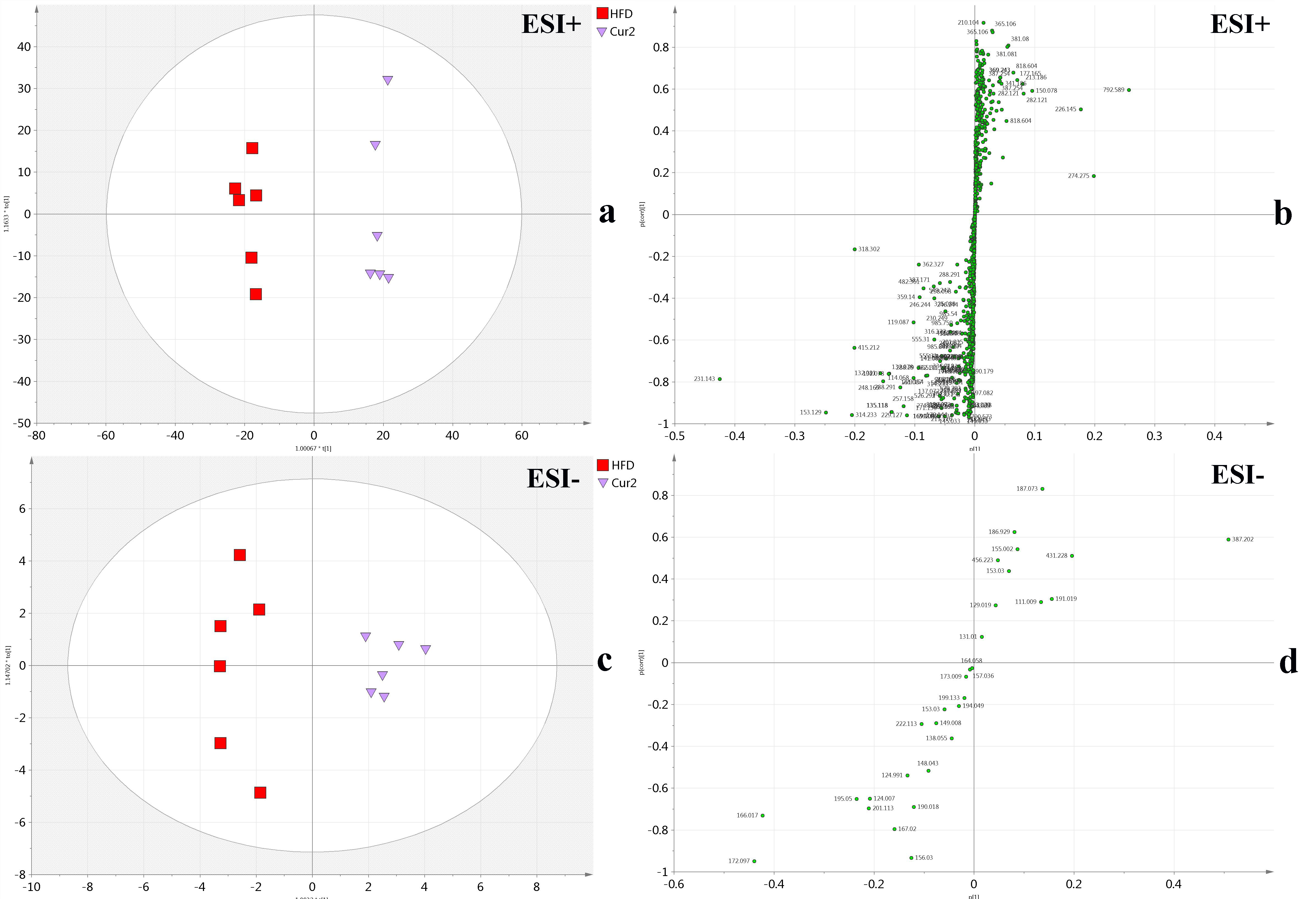

Supplement: S3 Fig — a, b for ESI+ mode and c, d for ESI- mode. (ESI+, R 2 Y = 0.987, Q 2 = 0.903; ESI-, R 2 Y = 0.942, Q 2 = 0.798). (TIF) [file pone.0120950.s003.tif]
