# Supplementary material for: 1H-NMR and MS Based Metabolomics Study of the Intervention Effect of Curcumin on Hyperlipidemia Mice Induced by High-Fat Diet
Source: PLoS One. 2015 Mar 18;10(3):e0120950. doi: 10.1371/journal.pone.0120950 (PMC4364983; doi:10.1371/journal.pone.0120950)
Supplement: S1 Table — (DOCX) [file pone.0120950.s004.docx]

**Table S1 Stability of UPLC-Q/TOF MS platform.**

Mode m/z t_R_ (min) Peak area t_R_(min) MW(Da)

Mean RSD (%) Mean Delta Mean Drift Value

ESI+ 149.1088 1.69 8853.84 9.27 1.69 0.02 149.1088 0.0007

ESI+ 330.0604 3.57 1699.70 8.83 3.57 0.06 330.0605 0.0010

ESI+ 915.7906 17.13 1531.31 9.73 17.13 0.01 915.7927 0.0080

ESI- 157.0366 0.81 523.55 5.16 0.81 0.02 157.0366 0.0015

ESI- 328.0453 3.56 780.57 9.68 3.56 0.05 328.0453 0.0050

ESI- 569.3356 17.63 3345.46 7.35 17.63 0.02 569.3356 0.0048
