# Supplementary material for: 1H-NMR and MS Based Metabolomics Study of the Intervention Effect of Curcumin on Hyperlipidemia Mice Induced by High-Fat Diet
Source: PLoS One. 2015 Mar 18;10(3):e0120950. doi: 10.1371/journal.pone.0120950 (PMC4364983; doi:10.1371/journal.pone.0120950)
Supplement: S2 Table — (DOCX) [file pone.0120950.s005.docx]

**Table S2 ^1^H-NMR assignment of the metabolites in urine of mice and related metabolic pathways.**

NO. Metabolites Moieties δ1H (ppm) and multiplicity Related pathway

1 1-methylnicotinamide 9.27 (s); 8.96 (d); 8.90 (d); Nicotinate and Nicotinamide Metabolism

2 Niacinamide 2-H, 6-H 8.98 (d); 8.70-8.71 (dd); Nicotinate and Nicotinamide Metabolism

3 Nicotinamide N-oxide 2-H, 6-H, 8.10-8.13 (d) Nicotinate and Nicotinamide Metabolism

4 Formate HCOO 8.46 (s) Folate Metabolism

5 Hippurate Ph-H 7.81-7.84 (d); 7.61-7.64 (m); 7.53-7.56 (t) Gut microbiome-derived metabolism

6 Benzoate Ph-H 7.46-7.49 (t) Hippurate synthesis

7 N-phenylacetylglycine Ph-H，NCH2CO 7.39-7.43 (m);7.33-7.37 (m); 3.36-3.37 (s) Metabolites of fatty acids

8 trans-Aconitate C=CH-CO 6.59-6.60 (s)

9 Urea CO(NH2)2 5.7-6.0 (s) Urea Cycle

10 cis-Aconitate C=CH-CO 5.74 (t) TCA cycle

11 Allantoin CONHCO, COCHN 6.09 (s); 5.37-5.38 (s) Metabolites Related to oxidative stress and kidney damage

12 glucose CH 4.66 (d) Glycolysis and gluconeogenesis

13 Tartrate COCOH-H 4.34-4.35 (s)

14 Creatinine N-CH3, N-CH2- 4.05 (s); 3.04 (s) Creatine metabolism

15 Creatine N-CH3, N-CH2- 3.92 (s); 3.02 (s) Creatine metabolism

16 Creatine phosphate N-CH3, N-CH2- 3.94 (s); 3.04 (s) Creatine metabolism

17 Taurine N-CH2, S-CH2 3.25-3.30 (t); 3.40-3.44 (t) Bile acid biosynthesis and taurine metabolism

18 Choline N(CH3)3 3.18-3.19 (s) Choline metabolism

19 Trimethylamine N(CH3)3 2.86 (s) Gut microbiome-derived metabolism

20 Dimethylamine N(CH3)2 2.70-2.71 Gut microbiome-derived metabolism

21 Citrate Half CH2, Half CH2 2.65-2.70 (d); 2.20-2.55 (d) TCA cycle

22 Methylamine NCH3 2.59-2.60 (s) Gut microbiome-derived metabolism

23 Succinate (COCH2)2 2.40 (s) TCA cycle

24 Pyruvate COCH3 2.37 (s) TCA cycle, glycolysis and gluconeogenesis

25 Acetoacetate COCH3 2.28 (s) Synthesis and degradation of ketone bodies

26 Acetone CO(CH3)2 2.22 (s) Synthesis and degradation of ketone bodies

27 Acetate COCH3 1.92 (d) Fatty acid oxidation

28 Alanine βCH3 1.49-1.49 (d) Glycolysis and gluconeogenesis

29 Lactate βCH3 1.33 (d) Glycolysis and gluconeogenesis

30 Valine γCH3 1.04 (d); 0.98 (d) Valine, leucine and isoleucine biosynthesis

31 Leucine /isoleucine δCH3; γCH3 0.98 (d); 0.94 (d) Valine, leucine and isoleucine biosynthesis

Note: s=singlet; d=doublet; dd=double doublet; t=triplet; q=quartet; m=multiplet.

1, 1-methylnicotinamide; 2, Niacinamide; 3, Nicotinamide N-oxide; 4, Formate; 5, Hippurate; 6, Benzoate; 7, N-phenylacetylglycine; 8, trans-Aconitate; 9, Urea; 10, cis-Aconitate;11, Allantoin; 12, Glucose; 13, Tartrate; 14, Creatinine; 15, Creatine; 16, Creatine phosphate; 17, Taurine; 18, Choline; 19, Trimethylamine; 20, Dimethylamine;21, Citrate; 22, Methylamine; 23, Succinate; 24, Pyruvate; 25, Acetoacetate; 26, acetone; 27, Acetate; 28, Lactate; 29, Leucine / isoleucine; 30, Valine; 31, Alanine
