# Supplementary material for: 1H-NMR and MS Based Metabolomics Study of the Intervention Effect of Curcumin on Hyperlipidemia Mice Induced by High-Fat Diet
Source: PLoS One. 2015 Mar 18;10(3):e0120950. doi: 10.1371/journal.pone.0120950 (PMC4364983; doi:10.1371/journal.pone.0120950)
Supplement: S3 Table — (DOCX) [file pone.0120950.s006.docx]

**Table S3** Potential biomarkers identified by MS, as well as chromatographic retention time, measured molecular mass, and related pathway.

Metabolites TR M/Z Adduct Delata Adduct ESI VIP Metabolic Pathway

(min) (D) (D) (D) Form mode

Palmitoylglycine 17.80 314.233 314.2617 -0.0287 [M+H]^+^ + 4.85 Fatty acid metabolism

Creatine^a^ 0.81 132.081 132.0694 0.0116 [M+H^]+^ + 3.85 Creatine metabolsim

Creatinine^a^ 0.84 114.068 114.0589 0.0091 [M+H]^+^ + 3.07 Creatine metabolsim

Glucose^a^ 17.52 181.087 181.0634 0.0236 [M+H]^+^ + 2.74 Gluconeogenesis

Succinic acid^a^ 18.61 119.0577 119.0339 0.0238 [M+H]^+^ + 2.39 TCA cycle

Acetylcarnitine 0.86 204.135 204.1230 0.0112 [M+H]^+^ + 1.70 Fatty acid metabolism

3-Hexenedioic

acid 17.83 145.033 145.0495 0.0165 [M+H]^+^ + 1.43 Fatty acid metabolism

Cysteine^a^ 5.27 122.03 122.0270 0.0030 [M+H]^+^ + 1.03 Taurine synthesis

Cysteine^a^ 0.84 166.017 166.0180 0.0009 [M+FA-H]^-^ - 1.42 Taurine synthesis

Taurine^a^ 0.79 124.007 124.0074 0.0004 [M-H]^-^ - 1.23 Bile Acid Biosynthesis

Note: HFD, Con, Cur1, Cur2, Lov represent hyperlipidemia, control, curcumin (40mg/kg), curcumin (80mg/kg), lovastin (30 mg/kg) groups, separately. ^a^ identified by reference standards.
